# Wuwei Qingzhuo San Ameliorates Hyperlipidemia in Mice Fed With HFD by Regulating Metabolomics and Intestinal Flora Composition

**DOI:** 10.3389/fphar.2022.842671

**Published:** 2022-06-27

**Authors:** Shasha Ge, Cuiping Liao, Duna Su, Tunuo Mula, Zhula Gegen, Zhiyong Li, Ya Tu

**Affiliations:** ^1^ Experimental Research Center, China Academy of Chinese medical sciences, Beijing, China; ^2^ Development Research Center of TCM, China Academy of Chinese Medical Science, Beijing, China; ^3^ Chi Feng an Ding Hospital, Chifeng, China; ^4^ College of Mongolian Medicine and Pharmacy, Inner Mongolia Minzu University, Tongliao, China; ^5^ Institute of Chinese Materia medica, China Academy of Chinese medical sciences, Beijing, China

**Keywords:** hyperlipidemia, Wuwei Qingzhuo San, metabolomics, 16S rRNA gene sequencing, intestinal microbiota

## Abstract

Hyperlipidemia is one of the most common metabolic disorders that threaten people’s health. Wuwei Qingzhuo San (WQS) is a traditional Mongolian medicine prescription, which is widely used in Mongolia for the treatment of hyperlipidemia. Our previous studies found that it has hypolipidemic and hepatoprotective effects on hyperlipidemic hamsters. However, the underlying lipid-lowering mechanisms of WQS and its relationship with intestinal flora are not yet clear. In this study, 16 S rRNA gene sequencing and metabolomics were performed to investigate the action mechanism of WQS on hyperlipidemic mice induced by a high-fat diet (HFD). As a result, metabolic pathway enrichment analysis revealed that the intervention of WQS had obviously modulated the metabolism of α-linolenic acid and linoleic acid and the biosynthesis of bile acids. 16 S rRNA sequencing showed that WQS had altered the composition of the intestinal microbiota in hyperlipidemic mice fed with HFD and, especially, adjusted the relative abundance ratio of Firmicutes/*Bacteroides*. These findings provide new evidence that WQS can improve HFD-induced hyperlipidemia by regulating metabolic disorders and intestinal flora imbalance.

## 1 Introduction

Hyperlipidemia is a condition that incorporates various acquired and inherited diseases, which have been described as an elevated level of lipid in the body (Hill and Bordoni, 2021). It is also considered a high-risk factor for atherosclerosis plaque and vascular disease that may ultimately lead to death (Mach et al., 2019). The incidence of hyperlipidemia in Chinese adults reaches 40% (Committee, C. A. D. G. R. J., 2016), and more than 3 million adults in the United States and Europe have been diagnosed with hyperlipidemia. Therefore, it is necessary to make early diagnoses and prevention in order to reduce morbidity and mortality.

Wuwei Qingzhuo San (WQS) is a traditional Mongolian prescription with a long clinical history (Bai, 1992), which was first recorded in the “BaiFang Chapter.” This prescription is composed of five traditional Chinese medicines, including *Punica granarum* L. (shiliu), *Carthamus tinctorius* L. (honghua), *Wurfbainia vera (Blackw.)* Myristica fragrans Houtt.(doukou), Neolitsea cassia (L.) Kosterm (rougui), and *Piper longum* L (biba). It has the effect of “relieving stagnation to promoting stomach fire (开郁消食)” and “Eliminating Phlegm and Producing Essence (清浊生华)” (Commission, C. P., 2020), which can be used to treat indigestion induced by overeating, abdominal distension, and diarrhea or some metabolic disease. It was found that the five herbs of WQS contain tannins, alkaloids, flavonoids, organic acids, and other components (Kumar et al., 2011; Ge et al., 2021) which have good effects in anti-inflammatory, immunomodulatory, and metabolic regulation. Our previous studies found that WQS has an attractive hypolipidemic and hepatoprotective effect in the hyperlipidemic hamster (Li et al., 2020), and associated endogenous metabolites in biological fluids were analyzed qualitatively and quantitatively using metabolomics (Hu and Xu, 2014). But its mechanism of regulation of cholesterol metabolism remains to be studied. Therefore, this study will focus on serum metabolomics to elaborate on how WQS regulates lipid metabolism.

Interestingly, recent research has found gut microbiota can maintain the physiological functions of the intestine, regulate diet, and host metabolism, and reduce the occurrence of metabolic disorders, so it is considered to be a regulator of host metabolism (Wang et al., 2020). Accumulating literature indicates that intestinal flora is related to the occurrence of chronic metabolic disorders (Lazar et al., 2019). Moreover, these five herbs in WQS are raw material medicine, which is different from the traditional Chinese prescription that needs to go through traditional processing techniques such as water extracting, so WQS will firstly go through catabolism of intestinal flora after oral administration, following the small molecular compounds are absorbed into the blood. Therefore, the study of the relationship between WQS and the gut microbiota is of some significance for the study of the hypolipidemic mechanism of this prescription.

In our previous study, it was predicted that WQS increases cholesterol catabolism through the upregulation of CYP7A1 and inhibits hepatic HMGCR expression through the upregulation of p-AMPK, thereby inhibiting cholesterol synthesis and lowering blood lipids(Li et al., 2020). Therefore, we extended our previous study to detect endogenous metabolites in mouse serum by UPLC-QTOF/MS-based serum metabolomics to determine whether the lipid-lowering mechanism of WQS is related to endogenous substances. Meanwhile, hyperlipidemia is closely related to intestinal flora, and the imbalance of intestinal flora will lead to changes in endogenous substances. The regulatory effect of WQS on the gut microbiota was also investigated by using 16 S rRNA gene sequencing. These studies provide a more comprehensive understanding of the mechanism by which WQS mediates therapeutic effects in HFD-induced hypolipidemic mice by regulating intestinal flora.

## 2 Materials and Methods

### 2.1 Materials and Animals

Wuwei Qingzhuo San was purchased from FuXin Mongolian Medicine Co., Ltd., (SFDA approval number: Z21020300, Inner Mongolia, China). It consists of *Punica granarum* L., *Carthamus tinctorius* L., *Wurfbainia vera (Blackw.)* Myristica fragrans Houtt.(doukou), Neolitsea cassia (L.) Kosterm, and *Piper longum* L with the mixed proportion of the respective compounds being 8:4:1:1:1. According to the traditional record of Mongolian medicine habit, the drug’s raw materials are often crushed into a powder and taken with water. Hence, WQS (Batch number: 20200614) in this study was dissolved in 0.5% carboxymethyl cellulose (CMC) buffer solution and prepared as a drug suspension before use.

Thirty male apolipoprotein E-deficient (Apoe^−/−^) on a C57BL/6J background mouse (8-week-old, weighting 18–22 g) were provided from Beijing Vital River Laboratory Animal Technology Co., Ltd. (SCXK-2011-0011). The animal experiment was approved by the animal committee of Medical Experimental Center, China Academy of Chinese Medical Sciences.

### 2.2 Animal Administration

Thirty male apoe^−/−^ mice were caged in controlled conditions of temperature (22 ± 2°C) and relative humidity (60 ± 5%) with a 12 h light/dark cycle for 7 days. Ten mice were fed normal chow as a normal control group (NC group, *n* = 10). Other apoe−/− mice were fed a high-fat diet (10% lard, 10% sucrose, 0.2% cholesterol, 0.5% bile salts, and 79.3% standard chow) as the HFD model group (HFD model group, *n* = 20) throughout the experimental period. After 4 weeks of HFD feeding, blood samples were collected from the orbital vein. The kit of Nanjing Jiancheng Institute of Bioengineering (Nanjing, China) was used to quantitatively determine the content of TC, TG, HDL-C, and LDL-C in the serum. Thereafter, according to the levels of blood lipid, these 20 mice in the HFD model group were randomly divided into two groups as follows: mice fed a high-fat diet as the HFD group (*n* = 10), and mice fed a high-fat diet + WQS as the WQS group (0.598 g/kg/day, *n* = 10). All mice were sacrificed after 6 weeks of WQS administration, serum samples were collected after centrifugation at 3,000 rpm for 15min. Ceca contents were washed from the cecum with 1.0 ml of cold Milli-Q water. All samples were stored at -80°C before analysis.

### 2.3 Serum Metabolomic

#### 2.3.1 Sample Preparation

Serum metabolomic analysis was performed on all mice in each group due to take into account the individual differences of mice in each group. The 50 μL serum samples were mixed with 200 μL methanol-acetonitrile solution (2:1, v/v) and sonicated for 5 min, then incubated for 20 min at -20°C freezers, the incubation solution was then centrifuged for 10 min (14,000 rpm, -4°C), and 200 μL of supernatant was injected into the LC/MS system for analysis. Furthermore, equal aliquots of the processed supernatants from each sample as the quality control (QC) sample.

#### 2.3.2 UPLC-Q-TOF/MS Analysis

Chromatographic separation was performed on a 1,290 Infinity series UPLC System (Agilent Technologies) with a UPLC BEH C_18_ column (2.1 × 100 mm, 1.7 μm, Waters). The mobile phase consisted of 0.1% formic acid aqueous (mobile phase A), and 0.1% formic acid acetonitrile (mobile phase B), and the gradient elution program with a flow rate of 0.4 ml min^−1^ was listed as follows: mobile phase B maintaining at 5% (0.00–0.50 min), from 5 to 30% (0.50–1.50 min), from 30 to 60% (1.50–4.00 min), from 60 to 99% (4.00–5.00 min) and maintaining at 95% (5.00–7.50 min).

Mass spectrometry information of small molecules was collected by the Q-TOF-MS spectrometer with an ESI source in negative or positive ion mode. The following MS conditions were used: the capillary voltage and cone voltage were 2.0 kV and 40 V, the desolvation gas temperature and source temperature were 440 and 115°C, and the flow rate of desolvation gas and cone gas was 80 L/h and 50 L, respectively, and the scan range was 50–1,000 m*/z*. The system control and data analysis were performed by Waters Progenesis QI v2.2 (Nonlinear Dynamics).

#### 2.3.3 Data Processing and Multivariate Analysis

The original data of UPLC-Q-TOF-MS/MS were processed by Progenesis QI v2.2 (Nonlinear Dynamics from Waters) combined with the SIMCA program (version 14.0, Sweden). PLS-DA, OPLS-DA, and PCA were carried out. And the value of R^2^X or R^2^Y and Q^2^ were used to evaluate the quality of the model. By default, SIMCA was used for seven rounds of cross-validation throughout the experiment to determine the most reasonable number of endogenous components and prevent excessive model fitting. And the OPLS-DA results needed further confirmation by a permutation evaluation (200 times). The value of P determined by the student’s test was used to select potential biomarkers. The biomarkers were identified by Progenesis QI MetaScope software and compared with the Human Metabolome Database (http://hmdb.ca/) based on accurate mass and specific MS^2^ fragments. Pathway enrichment analysis was performed on the MetaboAnalyst website (http://www.metaboanalyst.ca/) based on the KEGG database (Kyoto Encyclopedia of Genes and Genomes, http://www.kegg.jp/kegg/pathway.html).

### 2.4 Intestinal Flora Analysis

Metagenomic DNA from the contents in the cecum was extracted by MagPure Soil DNA LQ Kit (TransGen Biotech, Beijing, China) according to the manufacturer’s protocols. The bacteria DNA concentration and purity were estimated by a Nanodrop NC2000 spectrophotometer (Thermo Fisher Scientific Inc., USA). The hypervariable V3 -V4 region of bacteria 16 S rDNA genes was amplified by PCR with the primers (338 F: 5′-ACT​CCT​ACG​GGA​GGC​AGC​A) and primers (806 R: 5′GGACTACHVGGGTWTCTAAT). PCR was conducted using the following program: 98°C for 5 min, 24 cycles for 30 s at 98°C, 52°C for 30 s, 72°C for 45 s, 72°C for 5 min on an Eppendorf thermocycler. Amplification was confirmed by 2% agarose gel electrophoresis, PCR products were purified by the VAHTSTM DNA Clean Beads (Vazyme, Nanjing, China) and were quantified by the Quant-iT PicoGreen dsDNA Assay Kit on a quantitative Microplate reader (BioTek, FLx800). The V3-V4 region of bacterial 16 S rRNA genes was sequenced with the NovaSeq 6,000 platform (Illumina, USA) according to the manufacturer’s specifications. In addition, the clustering sequences were binned into operational taxonomic units (OTUs) with a 97% similarity cutoff using Vsearch (v2.13.4) (Rognes et al., 2016). Alpha diversity metrics (Chao1, Observed species, Shannon, Simpson index) and beta-diversity metrics (unweighted UniFrac (Lozupone and Knight, 2005) were respectively estimated by QIIME2 and the BrayCurtis distance algorithm.

### 2.5 Statistical Analysis

SPSS software (version 16.0) was used to analyze the data by one-way ANOVA and expressed as mean ± SD. Tukey’s multiple comparison test was used to identify significant treatment differences. Spearman correlation analysis was performed to determine the relationship between variables. *p* < 0.05 is considered to be statistically significant.

## 3 Results

### 3.1 WQS Administration Alleviates Hyperlipidemia in High-Fat-Diet Mice

**FIGURE 1:**
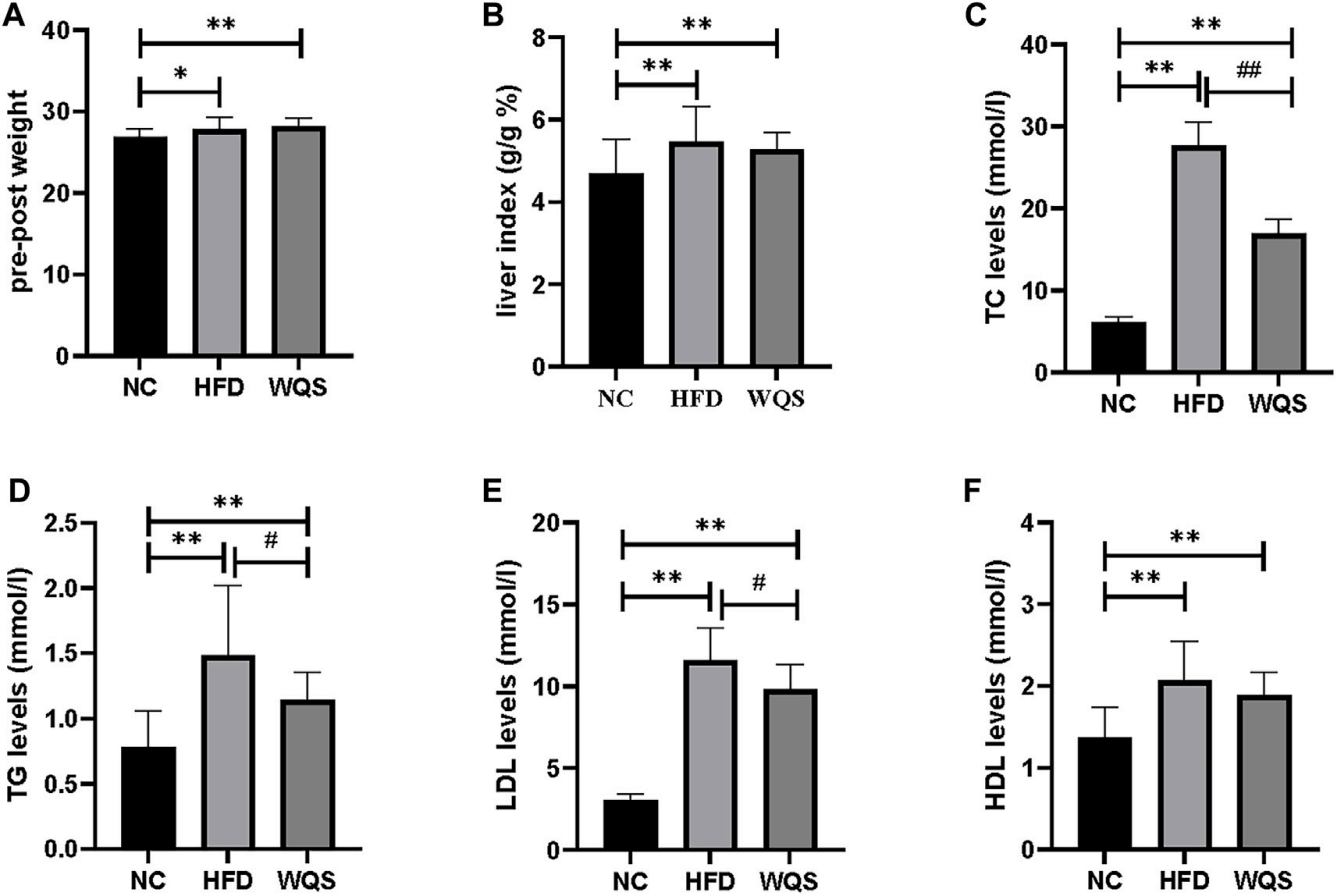
Effects of WQS on HFD-induced hyperlipidemia in mice. Relative change of **(A)** body weight; **(B)** liver index; **(C)** TC levels in serum; **(D)** TG levels in serum; **(E)** LDL-C levels in serum; **(F)** HDL-C levels in serum.

Male apoe^−/−^ mice developed hyperlipidemia after 10 weeks of HFD feeding. The weight (Figure 1A), liver index (Figure 1B), and blood lipid (Figures 1C–F) of mice with a high-fat diet were increased significantly, indicating that a diet-induced hyperlipidemia mice model was successfully established. After 6 weeks of WQS treatment, body weight and liver index were decreased, but there was no significant difference compared with the HFD group. In addition, the TC, TG, and LDL-c levels were markedly increased in the HFD group, but these were significantly decreased in the WQS group, especially on TC levels (*p* < 0.01). These results indicate that WQS effectively improved dyslipidemia in hyperlipidemia mice induced by continuous consumption of HFD.

### 3.2 WQS-Modulated Serum Metabolomic Profiling in HFD Mice

**FIGURE 2:**
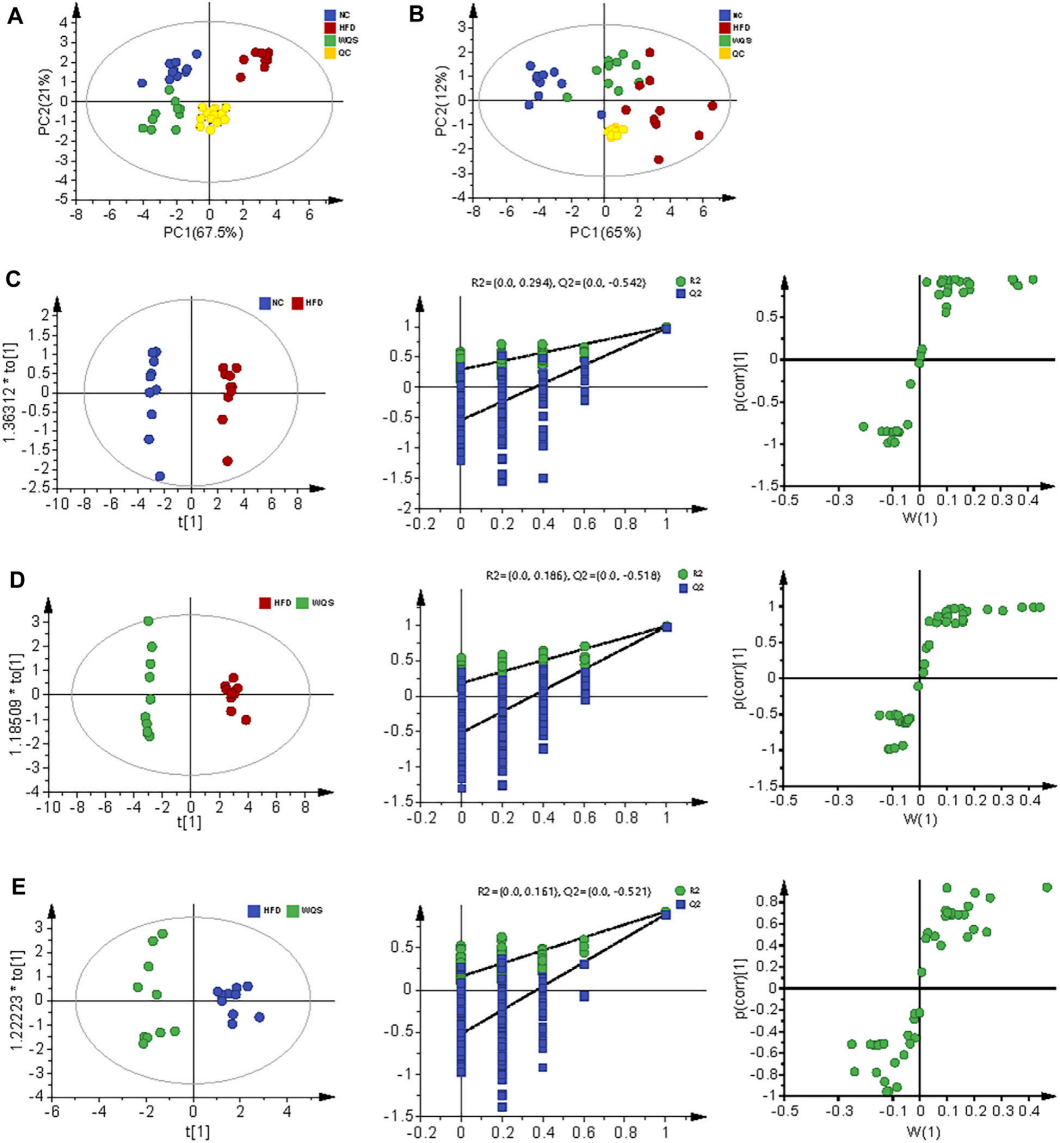
PCA for plasma from NC, HFD, and WQS groups in the positive **(A)** and negative **(B)** ESI mode. OPLS-DA score scatter plots, permutation test of OPLS-DA model, and S plots for NC group **(C)**, HFD group **(D)**, and WQS group **(E)** in the positive ESI mode.

**FIGURE 3:**
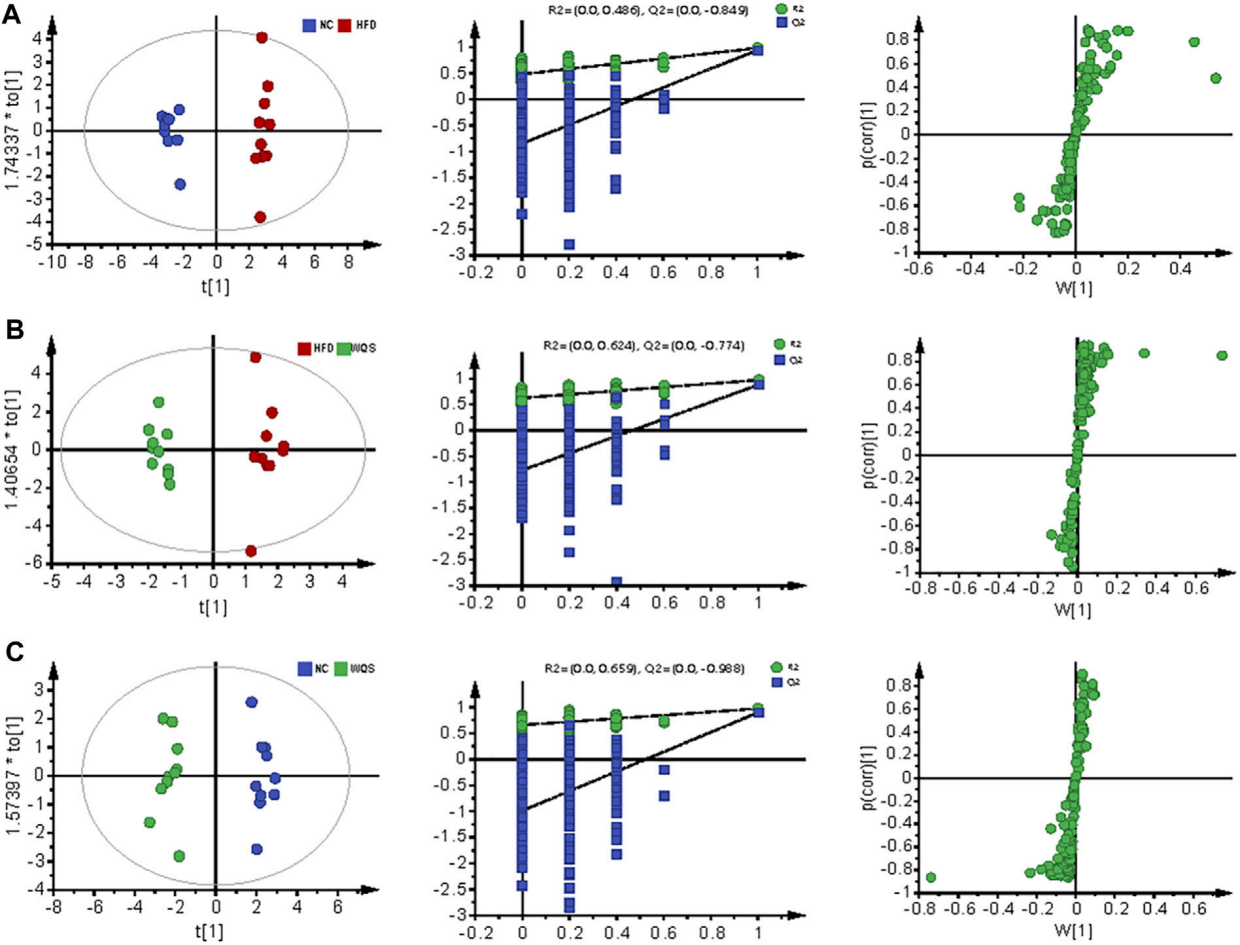
Serum metabolomic profiling by UPLC-QTOF MS in negative ion modes. OPLS-DA score scatter plots, permutation test of OPLS-DA model, and S plots for NC group **(A)**, HFD group **(B)**, and WQS group** (C)** in the negative ESI mode.

The representative base peak chromatogram (BPI) of serum samples from the NC, HFD, and WQS groups, and the BPI of QC samples in positive and negative ion modes are presented in Supplementary Figure S1 and Supplementary Table S1. The results of PCA showed that the three groups were clear separated in the positive and negative ion modes (Figures 2, 3). It indicates that serum biochemical disturbances occurred in hyperlipidemia mice, and the metabolic pattern changes significantly after oral administration of WQS. In addition, the WQS group was close to the NC group, which means that hyperlipidemia in mice showed the greatest improvement after oral administration of WQS (Figures 2A–B).

The score plot of OPLS-DA analysis showed obvious differences in metabolic characteristics among the groups, as shown in Figures 2C–E, especially the WQS group was distinguished from the HFD group. In addition, the permutation plot helped to obtain risk assessment of incorrect results from OPLS-DA. The 200 times permutation tests showed that all established OPLS-DA models are credible and have no over-fitting because the *R* ^2^ and Q^2^ values of the random permutation experiment were lower than the corresponding original values, and the regression line of Q^2^ had a negative intercept.

**FIGURE 4:**
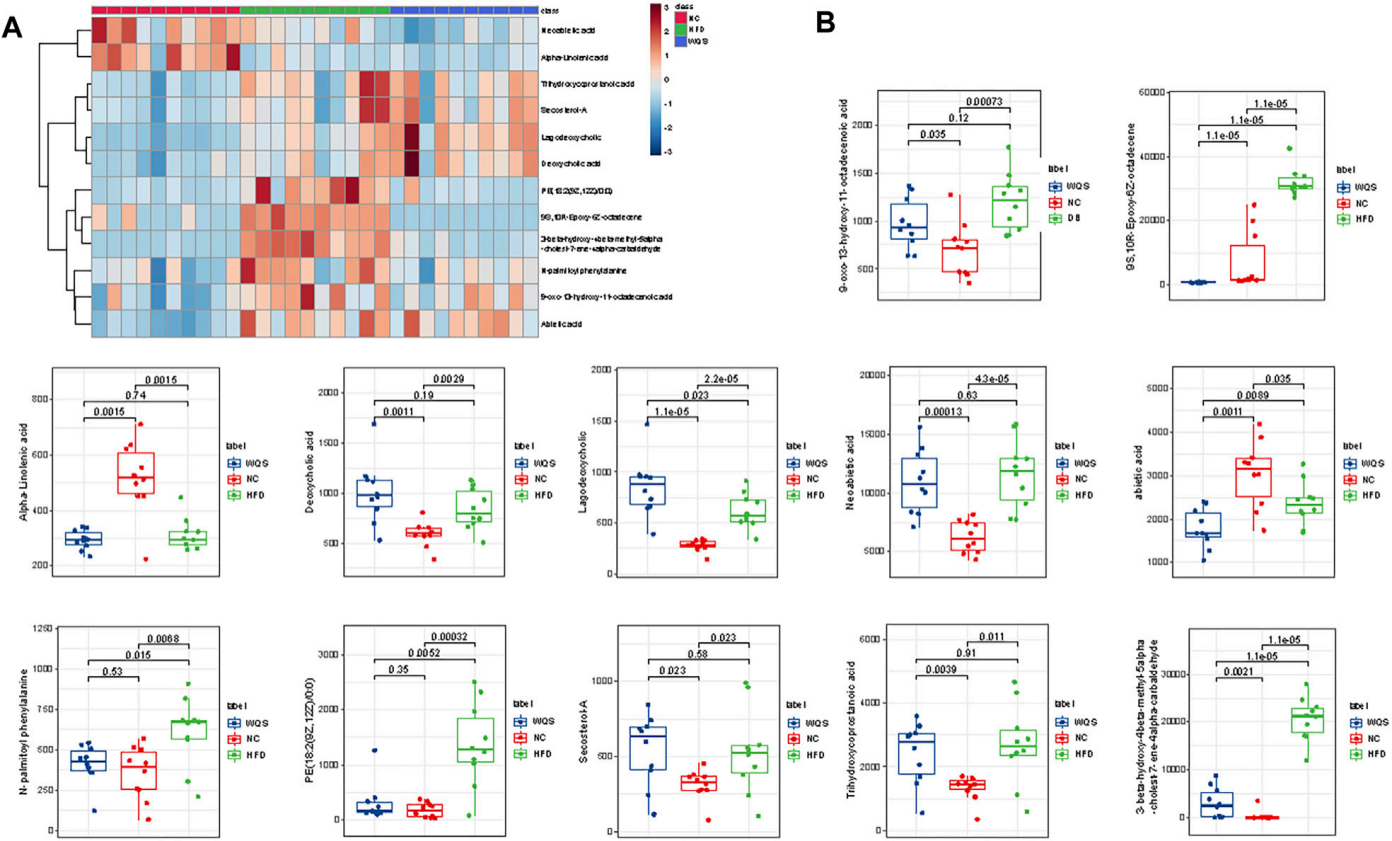
Heatmap to visualize the abundance of biomarkers in each group. **(A)** hierarchical clustering heat map of the 12 differential metabolites, with the degree of change marked with red (upregulation) and blue (downregulation); **(B)** 12 metabolites with differential abundance.

S- plot and ANOVA tests were conducted to reveal the potential biomarkers that contribute the most to the difference between groups. The points farthest from zero on the *X*-axis and *Y*-axis contributed the most to the difference between groups, and the metabolites with VIP> 1.5 and *p* < 0.05 are considered potential biomarkers (Figure 3). According to predefined criteria and the handling method, a total of 12 potential biomarkers were identified. The differences in the relative levels among the three groups were revealed by the cluster analysis of the heat maps of all metabolites, as shown in Figure 4A. Compared with the NC group, twelve metabolites were upregulated significantly in the HFD group, including 3-beta-hydroxy-4beta-methyl-5alpha-cholest-7-ene-4alpha-carbaldehyde, 9-oxo-13-hydroxy-11-octadecenoic acid, 9S-10 R-Epoxy-6Z-octadecene, deoxycholic acid, lagodeoxycholic, neoabietic acid, N-palmitoyl phenylalanine, PE (18:2 (9Z, 12Z)/0:0), secosterol-A, and trihydroxycoprostanoic acid. The levels of neoabietic acid-1, and Alpha-Linolenic acid were downregulated significantly. However, the levels of these metabolites in the WQS group were reversed and returned to normal or near to normal levels compared with the HFD group. Therefore, they were considered the potential biomarkers of the lipid-lowering effect of WQS. The Spearman correlation in these identified metabolites is shown in Figure 4B. In addition, the KEGG pathway annotation results of the metabolites showed that α-linolenic acid and linoleic acid metabolism and bile acid biosynthesis pathways were enriched, which might be possible pathways for WQS’s lipid-lowering.

### 3.3 WQS Supplementation Modulated Gut Microbial Community Composition

High-throughput 16 S rRNA sequencing combined with diversity analysis was used to investigate the influence of WQS on the intestinal microbial composition of HFD-fed mice. We drew the microbial classification tree based on the taxonomic annotations of species with OUT clustering and added the grouping abundance data of each taxa node to the graph in the form of a pie chart (Supplementary Figure S2A), and the threshold was set at the relative abundance of 0.5%, the taxa nodes whose relative abundance is greater than these thresholds at the same classification level.

The Alpha diversity results showed that a significant decrease in the richness of intestinal bacteria was observed in the HFD group measured by the Chao index and the observed species index compared to the NC group at the OTU level. However, the abundance of bacterial communities was increased after oral administration of WQS compared with the NC group (*p* < 0.01 in Supplementary Figure S2B). Meanwhile, the microbial community diversity of the HFD group decreased as measured by the Simpson and Shannon diversity index, but there was no statistical difference (Supplementary Figure S2B). The unweighted UniFrac analysis based on PCoA and NMDS analysis was conducted to compare similarities between intestinal microbial communities. The results revealed a notable separation of the microbial structure among the three groups (Supplementary Figures S2C,D). There was a significant difference among the three groups (R2 = 0.36, *p* = 0.001, adonis analysis), and observations of bacterial composition indicated that the high-fat diet altered the composition of the fecal microbiota, which had a considerable separation in microbial community with distance clustering to the NC group. Meanwhile, the microbial community of the WQS group was significantly separated from that of the HFD group, which indicated that WQS supplementation induced a remarkable change in gut microbial structure compared with the HFD group.

**FIGURE 5:**
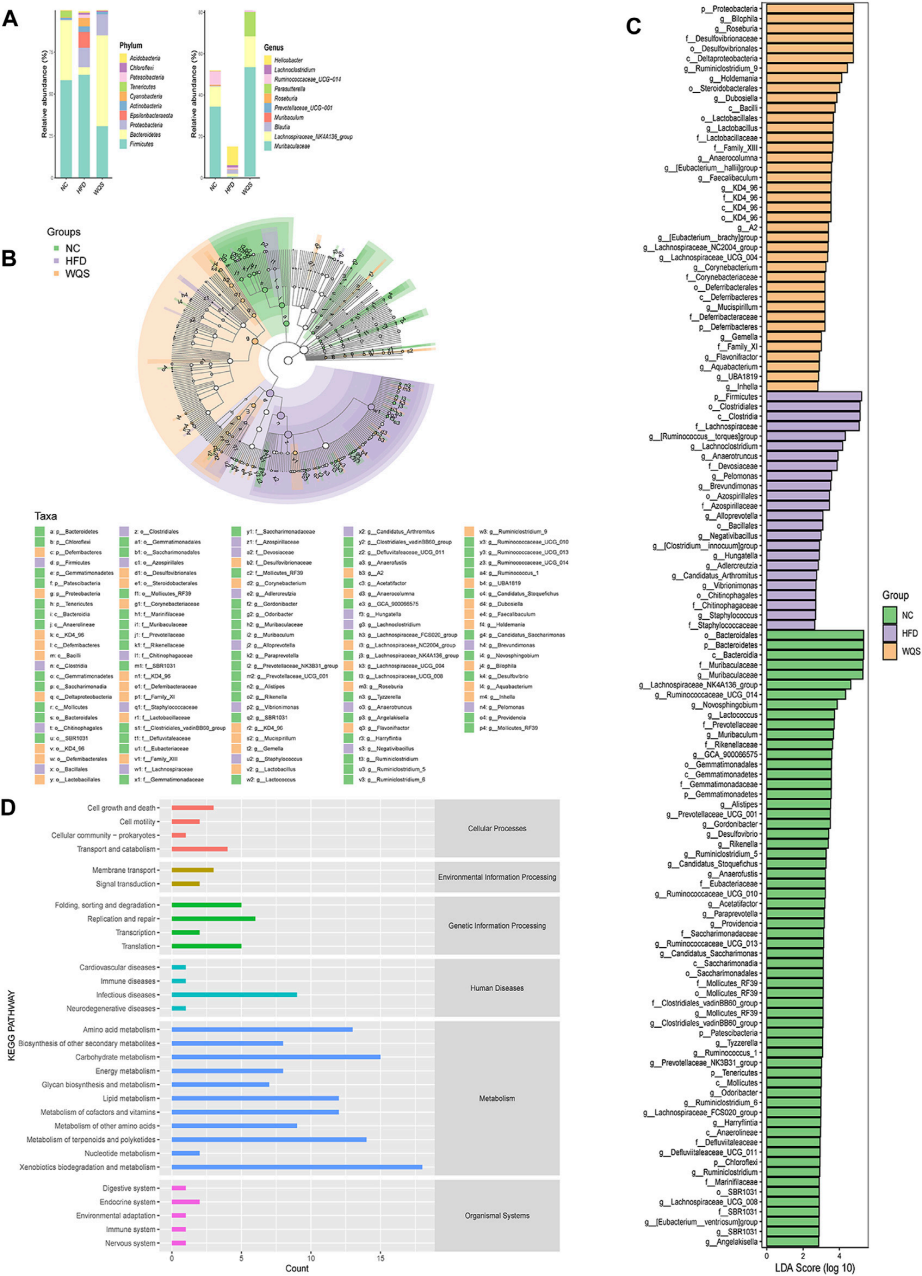
Effects of WQS on the changes of gut microbial composition and identification of most characteristic taxa among groups by linear discriminant analysis (LDA) effect size (LEfSe). **(A)** Composition of the microbial community at the phylum and genus level; **(B)** Most significant difference in gut microbial taxa between groups after LDA. The threshold on the logarithmic LDA score for discriminative features was set to 4.0. The length of the bar of the LDA represents the influence of species abundance on the different effects. **(C)** Cladogram visualizing the output of the LEfSe analysis. (c, class; f, family; g, genus; o, order; p, phylum). **(D)** KEGG pathway enrichment.

The results of OTUs statistical analysis showed that the structure of fecal bacteria was different among the three groups. As presented in Figure 5A, all three groups have four dominant phyla (Firmicutes, Bacteroidetes, Proteobacteria, and Actinobacteria) with different relative abundances. A significant rise in Firmicutes and a significant decline in *Bacteroides* and Proteobacteria were observed in the HFD group by comparing with the NC group. However, these changes were reversed after the oral administration of WQS. At the genus level, the relative abundance of Muribaculaceae, Blautia, [Eubacterium]_fissicatena_group, Lachnospiraceae_NK4A136_group, *Bacteroides*, and Ruminococcaceae_UCG-014_ was relatively lower, but the abundance of Bilophila, Roseburia, Lachnoclostridium, [Ruminococus]_torques_group, and Allobaculum was relatively higher in the HFD group.

To identify the most significant differences in specific gut microbial taxa from phyla to genera associated with different dietary interventions. LEfSe analysis was performed to analyze the microbial community. The results indicated that there are 125 rich differential taxa among the three groups (Figure 5B), (LDA>2, *p* < 0.05), including 70 genera. At the genus level, Muribaculaceae, Lachnospiraceae_NK4A136_group, Ruminococcaceae_UCG_014, and Novosphingobium and Lactococcus were dominant in the NC group. However, the HFD group was dominated by Rumencoccus __torques_group, Lachnoclostridium, Anaerotruncus, Pelomonas, and Brevundimonas. Therefore, the changes in these strains might be related to the pathogenesis of hyperlipidemia. The WQS group was dominated by Bilophila, Roseburia, Ruminiclostridium_9, Holdemania, and Dubosiella, these bacteria can be considered intestinal indicators for WQS to improve HFD-induced hyperlipidemia. The cladogram in Figure 5C further demonstrates the specific intestinal microbial taxa related to WQS treatment.

PICRUSt 2 (Phylogenetic Investigation of Communities by Reconstruction of Unobserved States) was used to study the changes in intestinal microbial function in HFD mice. Based on the KEGG database, PICRUSt displayed a total of six pathways of biological metabolism in Level 1 pathways (Figure 5D): cellular processes, environmental information processing, genetic information processing, human diseases, metabolism, and organismal systems. Metabolism and genetic information processing were dominant among them. At KEGG pathway level 2, it mainly included amino acid metabolism, carbohydrate metabolism, and metabolism of cofactor vitamin. HFD mice decreased 8 pathways and increased 12 pathways compared with the NC group.

### 3.4 Correlation Between Specific Microbial Taxa and Metabolic Parameters in Mice

**FIGURE 6:**
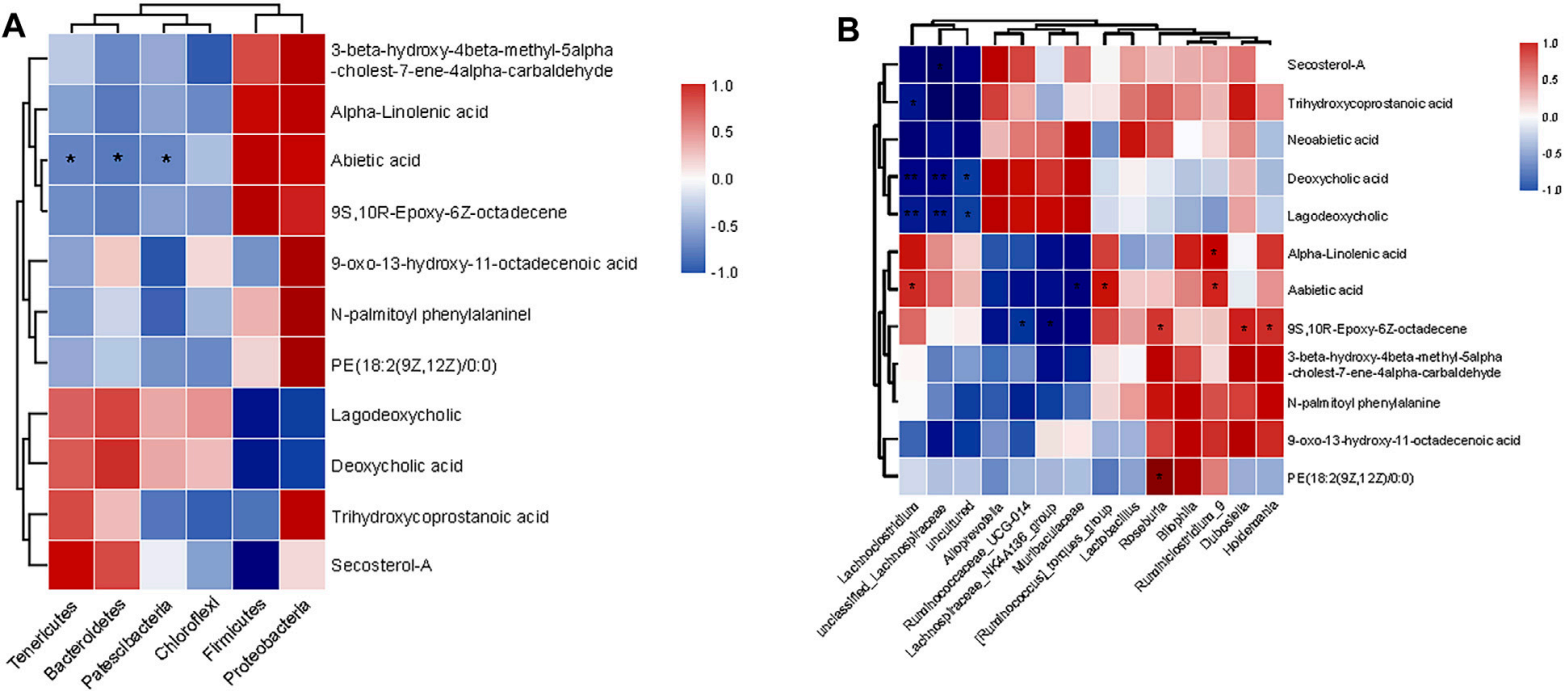
Spearman’s correlation between the identified metabolites and microbiota at different levels. **(A)** Correlation at the phylum level. **(B)** Correlation at the genus level.

To observe the relationship between metabolites and intestinal flora more intuitively, Spearman correlation analysis was performed on the three groups. At the phylum level (Figure 6A), Neoabietic acid showed negative relationships with Tenericutes, Bacteroidetes, and Patescibacteria. Similarly, at the genus level (Figure 6B), Secosterol-A, Trihydroxycoprostanoic acid, Deoxycholic acid, and Lagodeoxycholic showed negative relationships with Lachnoclostridium, unclassified_Lachnospiraceae, and uncultured_Lachnospiraceae. Meanwhile, Neoabietic acid-1 and 9 S, 10 R-Epoxy-6Z-octadecene showed negative relationships with Ruminococcaceae_UCG-014, Lachnospiraceae_NK4A136_group, and Muribaculaceae while positively related with Lachnoclostridium, [Ruminococcus]_torques_group, Roseburia, Ruminiclostridium_9, Dubosiella, and Holdemania. In addition, Alpha-Linolenic acid and PE (18:2 (9Z, 12Z)/0:0) correlated positively with Ruminiclostridium_9 and Roseburia.

## 4 Discussion

Hyperlipidemia is a potentially harmful disease, which will cause cardiovascular diseases with high mortality (Nelson, 2013). It will endanger people’s lives and health if patients do not receive timely medical treatment, bringing a heavy burden to the national medical insurance. At present, the main treatment of hyperlipidemia is a statin (Karr, 2017), but due to genetic and ethnic differences, while effective, it causes a markedly increased risk of myopathy (Sathasivam, 2012) and rhabdomyolysis (Antons et al., 2006). We still need to actively develop new drugs to deal with it. According to the records of traditional Mongolian medicine, the incidence of hyperlipidemia is due to excessive greasy ingredients in a daily diet and weak “stomach fire” (Zhang and Chang, 2015). Unable to complete normal metabolism to discharge unnecessary dregs, resulting in the continuous accumulation of greasy components in the blood. WQS can enhance the ability of “stomach fire” and “liver movement”, and improve the “metabolic power” of the human body (Hasgova, 2015), achieving the effect of cleaning up excess greasy components in blood lipids. Therefore, our study established a mouse model with hyperlipidemia (Burdge, 2006) and then orally administered WQS to observe changes in metabolism and intestinal bacteria in mice, and the relationship between them, revealing an underlying mechanism of alleviation of hyperlipidemia.

The hyperlipidemia mice model induced by HFD to simulate the clinical situation of patients with hyperlipidemia. ApoE−/− mice fed with a high-fat diet are indeed susceptible to hyperlipidemia were corresponding to previous reports (Fazio and Linton, 2001). By measuring the contents of blood lipids, including TC, TG, and LDL-C, we found that these index in mice fed with a high-fat diet was significantly increased compared with the control group, which proved that the hyperlipidemia mice model was established successfully. However, compared with those of the HFD group, TC and LDL-C levels decreased significantly in the WQS group. Moreover, TG levels decreased but there was no statistical difference. In particular, WQS has a significant decreased the level of TC, a typical hyperlipidemic indicator. All these results confirmed the lipid-lowering effect of WQS. It is worth mentioning that ellagic acid from *Punica granarum* (Ge et al., 2021), piperine and quercetin from *Piper longum* (Yadav et al., 2020) have been reported to have good lipid-regulating effects in WQS.

Through serum metabolomic analysis, we identified some metabolites associated with lipid metabolism in the serum of mice treated with WQS. The cluster analysis results showed that the main differential metabolite pathway was the α-linolenic acid and linoleic acid metabolism pathway in different groups. Linoleic acid (LNA), commonly referred to as omega-6 fatty acid, is a polyunsaturated fatty acid (PUFA) precursor of the longer n-6 fatty acid (Huang, 2006). PUFA α-linoleic acid (ALA) is also a precursor of n-3 fatty acids, called omega-3 fatty acids (Yue et al., 2020). Previous research reported that daily use of α-linolenic acid (ALA) can improve blood lipids in healthy non-obese men and women (Burak et al., 2019). Furthermore, the ALA diet improves blood lipid profile by reducing the levels of TG, TC, LDL, and VLDL-C in patients with hyperlipidemia or hyperglycemia. On the one hand, ALA can reduce the content of cholesterol in plasma and the liver by regulating cholesterol reverse transport (RCT) (Andersen and Fernandez, 2013). On the other hand, it can reduce cholesterol production by inhibiting the activity and mRNA expression of HMG-CoA reductase (Das, 2006). Furthermore, another report showed that ALA significantly decreases liver weight, liver cholesterol levels, and expression of cholesterol synthase (farnesyl pyrophosphate synthase) associated with hyperlipidemia (O'Reilly et al., 2020). There is some evidence that CLA promotes significant changes in HDL metabolism in the body, which has been shown to decrease plasma cholesterol levels and increase high-density lipoprotein levels in mice (den Hartigh, 2019). Moreover, CLA plays an important role in fat deposition in the liver and the development and improvement of insulin resistance (Moon et al., 2009). Interestingly, a report based on the foam cell model showed that CLA notably reduced the levels of both free and conjugated cholesterol, and the foam cell formation is via a PPARγ/LXRα-dependent regulation of cholesterol metabolism (Saini and Keum, 2018), which further confirms their specificity in atherosclerotic protection. Regulating the metabolism of these two fatty acids may be responsible for the effect of WQS on improving blood lipid status in hyperlipidemic mice.

As we all know, changes in the composition of the gut microbiota are closely related to some metabolic diseases, including hyperlipidemia and diabetes (He and You, 2020). In our results, the high-fat diet decreased intestinal bacteria diversity in mice, and the overall α-diversity was improved after WQS treatment in mice on the high-fat diet. Previous studies indicated that the diversity of gut microorganisms is beneficial to human health. The interaction between different types of intestinal flora can prevent certain strains of gut bacteria from reaching the level of manipulation of the host, thus further preventing the invasion of pathogenic bacteria. In our research, the diversity and composition of the microbiota in the HFD group have changed, with the Firmicutes increased and the *Bacteroides* and Proteobacteria decreased, which is similar to the situation of gut microbiota in obesity (Alcock et al., 2014). The increased abundance of Firmicutes is associated with the accumulation of lipid droplets, promoting fatty acid uptake at the onset of obesity and atherosclerosis (He and You, 2020). Roseburia (Tamanai-Shacoori et al., 2017), Ruminiclostridium (Jo et al., 2021), and *Lactobacillus* (Teng et al., 2020), as beneficial taxa, have high relative abundance in the WQS group, which might be a characteristic parameter that helps to significantly adjust blood lipids and relieve metabolic syndrome. Therefore, we deduced that these bacteria associated with the WQS supplement might be beneficial in decreasing excess cholesterol metabolism and adjusting lipid metabolism disorders in mice.

The balance of intestinal bacteria is closely related to the normal metabolic state of the body. Therefore, we performed serum metabolomics combined with Spearman analysis to observe the correlation of the gut microbiota with serum metabolites. Firmicutes and Proteobacteria displayed negative correlations with ALA, and Bacteroidetes showed positive correlations with ALA. It means that the content of ALA increases with decreasing Firmicutes/Bacteroidetes (F/B) ratio, which may help reduce the effect of a high F/B ratio, such as hyperlipidemia and obesity (Stojanov et al., 2020). Similarly, at the genus level, many genera are strongly correlated with differential metabolites, indicating that intestinal bacteria are indeed involved in the regulation of remodeling metabolism with the WQS intervention. Thus, we deeded that the regulation of blood lipids by WQS is related to the gut microbiota, and changes in the gut microbiota cause fluctuations in the content of endogenous metabolites in hyperlipidemic mice. However, intestinal bacteria could not be identified accurately and adequately by 16 S rRNA sequencing at the species level based on the Illumina platform, and the active constituent of WQS is not clear. Further studies are required to analyze the active compound of WQS and its relationship with key microbial phylotypes and lipid metabolic parameters, and to clarify their biological activities and action mechanism.

## 5 Conclusion

In this study, we found that a high-fat diet in ApoE^−/-^ mice can induce hyperlipidemia, the key strains of bacteria in the gut and serum metabolite had changed dramatically. In HFD-induced hyperlipidemic mice after the oral administration of WQS, their serum lipid profiles had shown improvement and the structure of the gut microbiota had been reshaped. There are thirteen endogenous metabolites in serum that were screened as biomarkers for regulating blood lipids, which have been associated with the alterations of the gut microbiota population. This study made us realize how the key intestinal bacteria strains and important metabolic biomarkers played a profound role in the procession of hyperlipidemia and provided rewarding information to discover new drugs for the treatment and prevention of hyperlipidemia. Although the lipid-lowering effect of WQS has been reported previously (Bai et al., 2017), it is to explain its underlying pharmacological mechanism from the insight of metabolomics and to explore the relationship of the intestinal flora with WQS in the treatment of hyperlipidemia for the first time. The finding of this research is helping further to explore the in-depth molecular mechanisms and promote WQS clinical application.

## Data Availability

The datasets presented in this study can be found in online repositories. The names of the repository/repositories and accession number(s) can be found at: NCBI with BioProject ID PRJNA812319.
