# Supplementary material for: Wuwei Qingzhuo San Ameliorates Hyperlipidemia in Mice Fed With HFD by Regulating Metabolomics and Intestinal Flora Composition
Source: Front Pharmacol. 2022 Jun 27;13:842671. doi: 10.3389/fphar.2022.842671 (PMC9272022; doi:10.3389/fphar.2022.842671)
Supplement: Supplementary file 1 [file DataSheet1.docx]

**Supplementary Material**

**Wuwei Qingzhuo San Ameliorates Hyperlipidemia in Mice Fed with HFD by Regulating Metabolomics and Intestinal Flora Composition**

Shasha Ge^1, 2 +^, Cuiping Liao^1, 2 +^, Duna Su^3^, Mula Tunuo^4^, Zhula Gegen^4^, Zhiyong Li^5^, Ya Tu^*1, 2^

1 Medical Research Center, China Academy of Chinese Medical Science, Beijing, China

2 Development Research Center of TCM, China Academy of Chinese Medical Science, Beijing, China

3 Chi Feng An Ding hospital, Chifeng, Inner Mongolia, China

4 School of Pharmacy, Inner Mongolia University for Nationalities, Tongliao, Inner Mongolia, China

5 School of Pharmacy, Minzu University of China, Beijing, China

Corresponding author

*Ya Tu, Development Research Center of TCM, China Academy of Chinese Medical Science, Beijing, China

Add: No. 16, Dongzhimen neinan street, Dongcheng district, Beijing, China, 100010

Tel: +086-010-64089691; Email: Tuya126@126.com

**Contents:**

**Figure 1**. Typical negative (right) and positive (left) base peak in serum patterns of BPI chromatograms in different groups.

**Figure 2**. Effects of WQS on the gut microbial composition in HFD-induced hyperlipidemic mice.

**Table 1.** Statistical analysis of Alpha diversity index


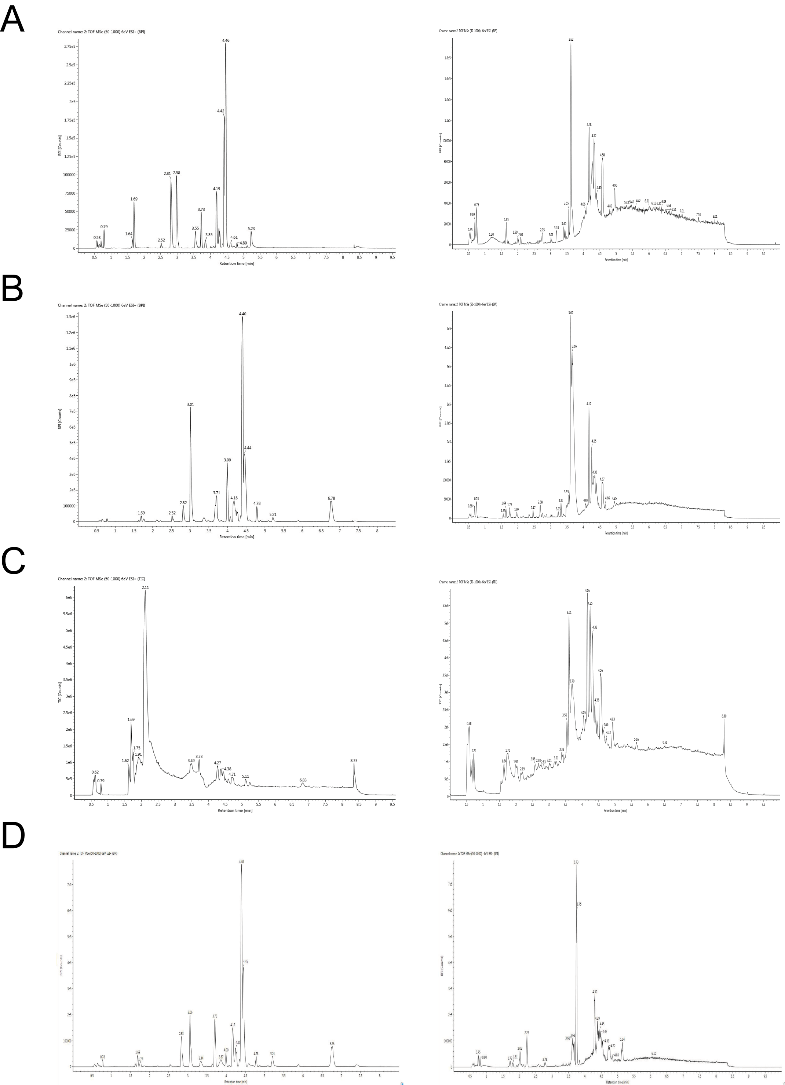


Figure 1. Typical negative (right) and positive (left) base peak in serum patterns of BPI chromatograms in different groups. (A) NC group; (B) HFD group; (C) WQS group; (D) QC samples.


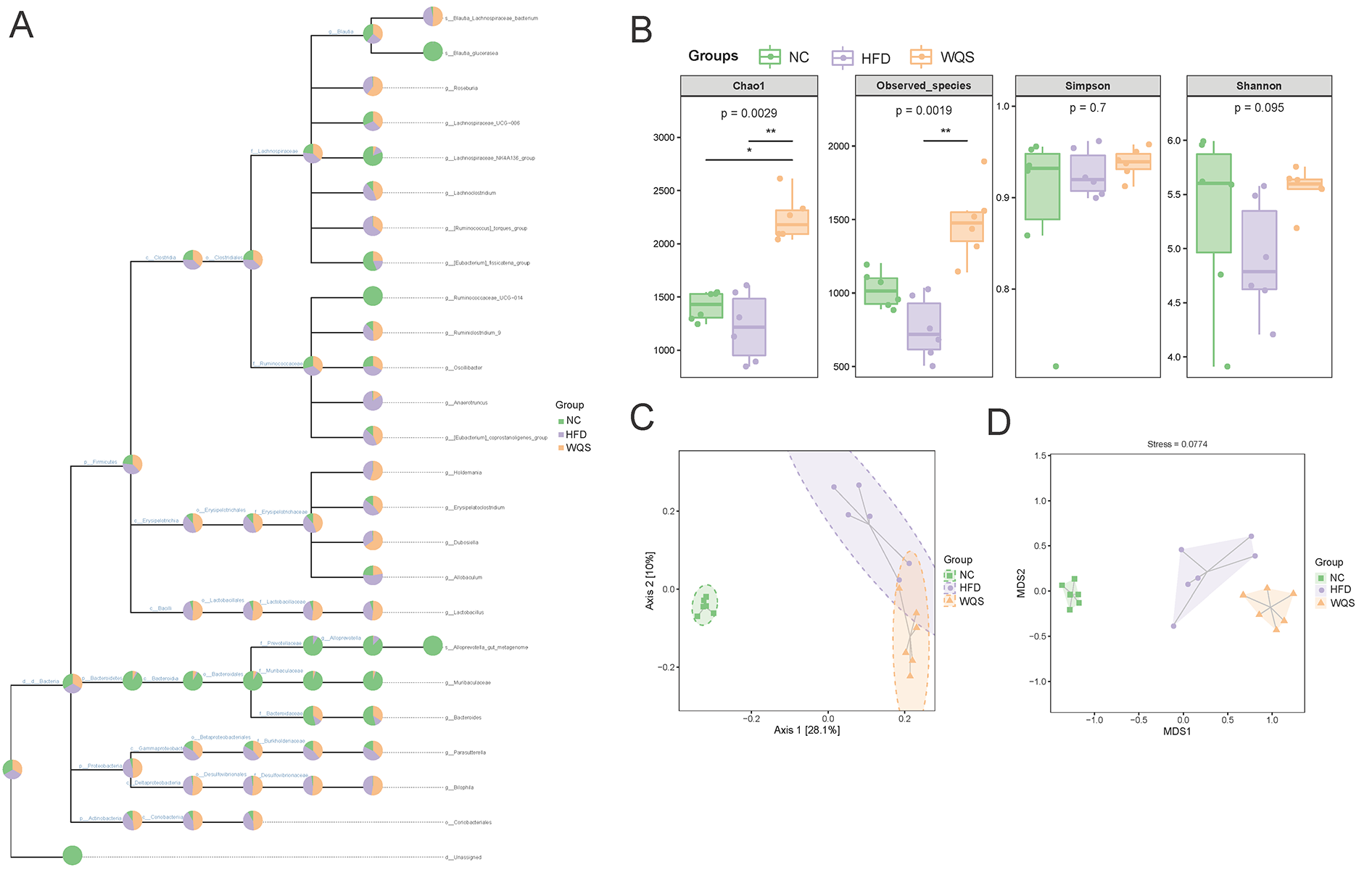


Figure 2. Effects of WQS on the gut microbial composition in HFD-induced hyperlipidemic mice.

1. Venn diagrams; (B) Chao1, Observed species, Shannon and Simpson indexes; (C) Unweighted UniFrac based on principal coordinate analysis (PCoA) with ellipse; (D) Unweighted UniFrac based on principal coordinate analysis (PCoA) with hull. *p<0.05, **p<0.01 among the three groups.

Table 1. Statistical analysis of Alpha diversity index

| **Sample ID** | **Chao1** | **Observed species** | **Simpson** | **Shannon** |
| --- | --- | --- | --- | --- |
| NC_1 | 1127.36 | 685.7 | 0.954119 | 5.48993 |
| NC_2 | 892.505 | 592.9 | 0.917316 | 4.658 |
| NC_3 | 846.363 | 506.1 | 0.903884 | 4.2071 |
| NC_4 | 1307.9 | 750.4 | 0.961554 | 5.57843 |
| NC_5 | 1542.41 | 989.7 | 0.899512 | 4.61313 |
| NC_6 | 1609.68 | 1035.1 | 0.921884 | 4.91794 |
| HFD_1 | 1244.74 | 949.5 | 0.955567 | 5.95494 |
| HFD_2 | 1333.91 | 917.6 | 0.858529 | 4.7569 |
| HFD_3 | 1529.43 | 1108.3 | 0.934743 | 5.58685 |
| HFD_4 | 1526.58 | 1078.8 | 0.929315 | 5.6205 |
| HFD_5 | 1295.49 | 891.1 | 0.715229 | 3.9119 |
| HFD_6 | 1544.34 | 1202.2 | 0.952447 | 5.99253 |
| WQS_1 | 2330.21 | 1512.4 | 0.937414 | 5.63326 |
| WQS_2 | 2267.56 | 1562.2 | 0.94083 | 5.75546 |
| WQS_3 | 2091.3 | 1321.7 | 0.958037 | 5.64178 |
| WQS_4 | 2611.16 | 1896.8 | 0.912305 | 5.54689 |
| WQS_5 | 2091.84 | 1441.6 | 0.949997 | 5.55968 |
| WQS_6 | 2040.69 | 1141.3 | 0.928913 | 5.18941 |
